# Correlates of Moderate-to-Vigorous Physical Activity in Children With Physical Illness and Physical–Mental Multimorbidity

**DOI:** 10.1177/10901981221100697

**Published:** 2022-06-13

**Authors:** Chloe Bedard, Sara King-Dowling, Joyce Obeid, Brian W. Timmons, Mark A. Ferro

**Affiliations:** 1 University of Waterloo, Waterloo, Ontario, Canada; 2 The Children’s Hospital of Philadelphia, Philadelphia, PA, USA; 3 McMaster University, Hamilton, Ontario, Canada

**Keywords:** accelerometry, socio-ecological model, children

## Abstract

This study measured physical activity (PA) and explored its correlates among children with multimorbidity (co-occurring chronic physical and mental illness; MM) versus those with chronic physical illness only (PI). This study used baseline data from the Multimorbidity in Children and Youth Across the Life Course (MY LIFE) study, an on-going cohort study following 263 children with a PI 2 to 16 years of age (mean age: 9.8 years, *SD* = 4.0; 47.7% female). PA was measured using accelerometry, and demographic and psychosocial variables were collected using questionnaires. Of the 55 children with MM and the 85 with PI with valid accelerometer data, 38.1% and 41.2%, respectively, met average daily PA guidelines. Correlates of moderate-to-physical PA (MVPA) among children with MM were age, ρ(53) = −0.45, *p* = .001, body mass index (BMI), ρ(48) = −0.28, *p* = .04, self-perceived behavioral conduct, ρ(24) = −0.45, *p* = .02, physical health-related quality of life, ρ(51) = 0.56, *p* < .001, and peer support, ρ(52) = 0.27, *p* = .04. Correlates of MVPA among children with PI were age, ρ(83) = −0.40, *p* < .001, sex, ρ(83) = −0.26, *p* = .01, self-perceived social competence, ρ(31) = 0.42, *p* = .02, self-perceived athletic competence, ρ(31) = 0.48, *p* = .005, physical health-related quality of life, ρ(83) = 0.34, *p* = .001, participation in community sport, ρ(31) = 0.41, *p* = .02, and family functioning, ρ(83) = 0.26, *p* = .02. These results demonstrate that children with PI and MM are insufficiently active and their PA is correlated with demographic and psychosocial factors.

Approximately, 25% of children and adolescents have a chronic physical illnesses such as asthma, juvenile arthritis, diabetes, epilepsy, or cerebral palsy, which increases their risk of experiencing physical, psychosocial, and emotional health challenges (Schwartz & Feudtner, 2019; van Cleave et al., 2010). Estimates suggest that 40% to 50% of these children frequently experience a co-occurring mental illness, known as multimorbidity (Butler et al., 2018; Ferro et al., 2022). Children with multimorbidity represent a proportion of a particularly vulnerable population who utilize a large proportion of the public health and the health care systems. Studies of high-cost users of the health system indicate that only 5% of children account for approximately 60% of health care costs, and these costs are mostly due to treatment of chronic illness (and often multimorbidity) (Wodchis et al., 2016). Furthermore, there is an increased rate of mental health care service use among children and youth (Gandhi et al., 2016), and while evidence is limited, it is possible that children with multimorbidity are disproportionately represented among mental health care users (Reaume et al., 2021).

While evidence of the complex health care needs of children with multimorbidity is growing, there remains important gaps in our understanding, particularly surrounding their engagement in physical activity (PA). Engagement in PA among children with chronic physical illnesses can improve aerobic capacity and internalizing symptoms (Tran et al., 2020) and has long been touted as a secondary disease prevention strategy (Durstine et al., 2013). Yet, children with chronic physical illnesses engage in insufficient levels of PA (Elmesmari et al., 2017). There is little known about the PA levels of children with multimorbidity—a critical gap in the literature. Children with multimorbidity face unique challenges (e.g., physical or bodily pain) and increased burdens to their health (e.g., decreased physical health-related quality of life, poorer social relationships, worse psychological well-being) compared with typically developing children and those with a physical illness only (Ferro, Qureshi et al., 2021; Tompke et al., 2020; Waters et al., 2008); therefore, it is possible that their engagement in PA is likewise differentially affected. Participation in PA has been linked to a plethora of physical health outcomes, such as improved physical fitness, bone mineral density, and cardiovascular health (Janssen & LeBlanc, 2010; Poitras et al., 2016). PA is also known to contribute to reduced symptoms of depression and anxiety and improved psychosocial well-being (Lubans et al., 2016; Poitras et al., 2016). Given both the promotive effects of PA on physical and mental health and the unique challenges experienced by children with multimorbidity, PA appears to be a particularly relevant and important element to understand in the context of childhood multimorbidity.

Relevant correlates of PA among children with multimorbidity are also unknown. Systematic reviews demonstrate that PA engagement is influenced by demographic, health, psychosocial, behavioral, and environmental factors (Sallis et al., 2000; Schmutz et al., 2017; Spence et al., 2010). PA in typically developing children is consistently related to being male, younger, greater perceived athletic or physical competence, more support from parents and peers, higher motor proficiency, engagement in community sports, warmer seasons, and easier access to facilities (Sallis et al., 2000; Schmutz et al., 2017). There has been comparatively less research on the influence of these factors in children with chronic physical and/or mental illnesses. Reviews on the correlates of PA among children with physical disabilities emphasize the unique influences of their physical limitations and health on participating in PA; however, psychosocial and environmental factors remain important (Li et al., 2016). Mangerud and colleagues (2014) found that PA among adolescents with psychiatric disorders was not explained by correlates of PA among the general population—sex, body mass index (BMI), and socioeconomic status. Rather, PA was explained by the type of psychiatric illness (e.g., mood vs. eating disorders). The discrepancy highlights the potential differences in correlates between children with and without multimorbidity, further supporting the need to examine the correlates of PA within this specific population of children.

Therefore, the objectives of this study were to:

1. Estimate levels of PA in children with multimorbidity (MM) and those with a physical illness only (PI); and
2. Identify demographic, health, psychological/cognitive/emotional, behavioral, familial, and environmental correlates of PA among children with PI and children with MM.

## Method

### Participants

This study used baseline data from the Multimorbidity in Children and Youth Across the Life Course (MY LIFE) study. MY LIFE is an on-going prospective study following 263 children ages 2 to 16 years attending outpatient clinics at an academic pediatric hospital in Canada to evaluate the trajectories of mental health in children with physical illness (Ferro et al., 2019). Eligibility required children to have a physician-diagnosed chronic physical illness (defined as a condition expected to last ≥12 months and lead to functional limitations, dependencies to compensate for limitations, and need for additional health care; Stein & Silver, 1999) and have parents with sufficient English language skills.

### Sampling Method

Participants were recruited from dermatology, endocrinology, gastroenterology, hematology, immunology, neurology, respiratory, and rheumatology subspecialty clinics. Descriptive statistics of key variables are provided in Table 1. Refer to Ferro, Lipman et al. (2021) for a full description of the cohort.

**Table 1.** Descriptive Statistics of the MY LIFE Sample With Valid Accelerometer Data at Baseline.

Variable	Multimorbidity (*n* = 55)	Physical illness only (*n* = 85)
Child’s age in years, *M* (*SD*)	9.9 (3.69)	8.89 (3.77)
Female, *n* (%)	20 (36.4%)	52 (61.2%)
Child’s BMI, *M* (*SD*)	19.06 (4.81)	18.13 (4.39)
Underweight, *n* (%)	1 (1.8%)	0 (0%)
Normal weight, *n* (%)	33 (60.0%)	60 (70.6%)
Overweight/obesity, *n* (%)	16 (29.1%)	21 (24.7%)
Child born in Canada, *n* (%)	52 (94.5%)	81 (95.3%)
Parent born in Canada, *n* (%)	48 (87.3%)	73 (85.9%)
Parent race		
White, *n* (%)	49 (89%)	73 (86%)
Latin American, *n* (%)	1 (2%)	2 (3%)
Black, *n* (%)	1 (2%)	1 (1%)
Arab, *n* (%)	1 (2%)	0 (0%)
South Asian, *n* (%)	1 (2%)	1 (1%)
Southeast Asian, *n* (%)	0 (0%)	1 (1%)
Other, *n* (%)	4 (7%)	7 (8%)
Parent relationship status (married or common law), *n* (%)	49 (89.1%)	72 (84.7%)
Household income <CAD$119,000, *n* (%)	32 (58.2%)	41 (48.2%)

*Note. SD* = standard deviations; *n* = sample size; BMI *=* body mass index.

### Study Procedures

Parents and youth ≥10 years of age completed computer-assisted self-report questionnaires, research assistants collected biological samples, and accelerometers were fitted to participants. Baseline study appointments were scheduled between August 2017 and November 2019 with follow-up appointments at 6, 12, and 24 months (data collection on-going). All participants ≥7 years provided written informed assent, and written informed consent was obtained from the parents of all enrolled participants and youth ≥16 years of age. MY LIFE received ethical approval from the Hamilton Integration Research Ethics Board.

### Measures

#### Multimorbidity

The Mini International Neuropsychiatric Interview for Children and Adolescents (MINI-KID) was used to identify children with multimorbidity. The MINI-KID is a diagnostic interview tool that aligns with the diagnostic criteria of both the *Diagnostic and Statistical Manual of Mental Disorders* (5th ed., *DSM*-*V*; American Psychiatric Association, 2013) and the *International Classification of Diseases, Tenth Revision* (ICD-10). Among parents of youth ages 9 to 18 years from both general and clinical populations, the test–retest (K = 0.71) and convergent validity (β = 0.67) were similar to other standardized diagnostic interviews (Boyle, Duncan et al., 2019; Duncan et al., 2018; McDonald et al., 2021). Eight MINI-KID modules were administered to measure major depressive episode, generalized anxiety disorder, separation anxiety disorder, social anxiety disorder, specific phobias, attention-deficit hyperactivity disorder, oppositional defiant disorder, and conduct disorder. The parent version of the MINI-KID was administered over the telephone prior to the first study appointment. Participants meeting the diagnostic criteria for any of the above mental illnesses were classified as having multimorbidity (MM) and those not meeting criteria for any mental illness will be considered as having a physical illness only (PI).

#### Physical Activity

Accelerometry is a device-based method of assessing free-living PA. Participants were fitted with an ActiGraph GT3X activity monitor secured by a belt worn around the waist, to be worn over their right hip for 7 days (Trost et al., 2000). Raw data collected at 30 Hz were downloaded and converted into 3 s epochs for analyses (Obeid et al., 2011). Parents were instructed to have their child wear the device during all waking hours except for water-based activities (i.e., showering, bathing, swimming) and were given a log to record wear time. Non-wear time for all participants was defined as any time the log book indicated the device was removed and/or when >60 minutes of consecutive zeros counts were detected (Colley et al., 2010; Troiano et al., 2008). To ensure that daily accelerometer data were reasonably representative (between day reliability of ~87%), a wear time criterion of ≥10 hours per day for ≥3 days was established (Rich et al., 2013). To examine correlates of PA across all age groups, the Evenson cut points were used to determine average time per day spent in moderate-to-vigorous physical activity (MVPA; Trost et al., 2011) for all children. Evenson cut points have strong evidence of accuracy when classifying MVPA intensities with area under the curve (AUC) ranging from 0.70 to 0.90 when applied as a single cut point across ages 5 to 15 years (Evenson et al., 2008; Trost et al., 2011). Pate cut points have good evidence of their validity when applied to both preschoolers and toddlers (Trost et al., 2012) and were therefore also applied to children ages 2 to 4 years to assess adherence to PA guidelines for the early years. All accelerometer data were cleaned and processed using the ActiLife Software (ActiGraph).

#### Demographic and Health Covariates

Parents were asked to report information about age, sex, immigration status (relating to both themselves and their child), race, as well as their relationship status and household income.

The World Health Organization Disability Assessment Schedule (WHODAS) 2.0 was used to assess health disability (Üstün et al., 2010). The 12-item WHODAS 2.0 asks parents to rate the level of difficulty their child experiences when engaging in specific activities within the domains of cognition, mobility, self-care, getting along, life activities and participation on a 5-point scale ranging from “none” (0) to “extreme or cannot do” (4). Scores were summed to create a total score with higher values reflecting greater functional limitations. The WHODAS 2.0 has evidence of partial measurement invariance in youth with physical or mental illness (Kimber et al., 2015; Tompke et al., 2020) and a track record of use in the study sample (Ferro et al., 2022).

Standing height and weight were measured and BMI (kg·m^-2^) percentiles were calculated based on the World Health Organization growth reference data for children (Onis et al., 2007).

#### Psychological/Cognitive/Emotional Covariates

The Strengths and Difficulties Questionnaire (SDQ) was used to assess emotional and behavioral problems for children ages 3 to 16 years (Goodman, 2001). The SDQ is a parent-report scale that assesses emotional symptoms, conduct problems, hyperactivity/inattention, peer relationship problems, and pro-social behaviors using 25 items and a 3-point scale. An externalizing score summed the conduct and hyperactivity scales and an internalizing score summed the emotional and peer problem scales, with higher scores indicating greater levels of problems. The factor structure and the test–retest reliability (mean correlation = 0.62) have been confirmed among parents of children age 5 to 15 years (Goodman, 2001).

The Self-Perception Profile for Children (SPPC) was administered to participants ≥10 years to assess their perceived scholastic competence, social competence, athletic competence, physical appearance, behavioral conduct, and global self-worth (Harter, 1982). Subscales were summed with higher scores reflecting more positive perceptions. The SPPC has been shown to be correlated to global self-concept and domains of quality of life, as well as internally consistent (α = 0.83–0.95) among children with a chronic illness (Ferro & Tang, 2017).

The parent-reported KIDSCREEN-27 was used to evaluate dimensions of health-related quality of life including physical well-being, psychological well-being, autonomy and parents, support, and school environment for children on a 5-point scale (Ravens-Sieberer et al., 2014). Raw scores were transformed into standard *T*-scores (mean of 50 and standard deviation of 10) for each subscale; higher scores indicate better health-related quality of life. It has robust evidence of reliability, validity, and sensitivity among children as young as 2 years of age with typical development and those with physical or mental illness in over 30 countries (Ferro, Otto, & Ravens-Sieberer, 2021; Qadeer & Ferro, 2018; Ravens-Sieberer et al., 2014; Tompke & Ferro, 2019).

#### Behavioral Covariates

Involvement in community sports was assessed using two items from the Ontario Child Health Study (OCHS) that asked participants (youth ≥10 years of age) how often they participated in organized and unorganized sport or PA outside of school in the past 12 months; response options range from “most days” to “almost never” (Boyle, Georgiades et al., 2019). Items scores were summed and ranged from 2 to 10, with higher scores indicating higher levels of participation.

#### Familial Covariate

Family functioning was assessed using the General Functioning subscale of the McMaster Family Assessment Device (FAD; Epstein et al., 1983). Twelve items ask parents to rate their agreement on a 4-point scale to statements that describe family functioning in relation to communication, problem solving, involvement, and control. Item scores were summed to create a total score with high values reflecting better family functioning. The FAD shows good reliability (Cronbach’s α = 0.86) and convergent and discriminant validity among families of children ages 4 to 16 years and has been used successful among families of children ages 8 to 17 years with chronic illnesses (Oltean et al., 2020).

#### Environmental Covariate

Seasonality was a categorical variable (winter, spring, summer, or fall) defined according to the National Research Council Canada and based on the first day the participant wore the accelerometer (National Research Council Canada, 2020).

### Missing Data

Of the 263 children participating in the study, 140 provided valid accelerometer data (≥10 hours of wear time on ≥ 3 days). Missing accelerometer data were not associated with a positive screen on the MINI-KID, nor with any other independent variable explored in the study. However, children ≥10 years of age were slightly more likely than children <10 years to have missing accelerometer data, with 50.4% and 43.8% missing data, respectively.

### Statistical Analyses

The proportion of children meeting PA guidelines was determined by age group. Children ages 5 to 16 years with an average daily MVPA of ≥60 minutes as defined using the Evenson cut points (Evenson et al., 2008) were considered to have met guidelines. Children 3 or 4 years engaging in an average daily PA of ≥180 minutes at any intensity and an average of ≥60 minutes of MVPA daily as defined by Pate cut points (Pate et al., 2006) were considered to have met the guidelines (Tremblay et al., 2016). Children <3 years engaging in an average daily PA of ≥180 minutes at any intensity as defined by Pate cut points (Pate et al., 2006) were considered to have met the guidelines (Tremblay et al., 2016). Bivariate correlational analyses were used to establish variables associated with average daily MVPA. Average minutes per day spent in MVPA was computed as sum of the minutes of MVPA on every valid day of wear divided by the number of valid days of wear. Time spent in MVPA was defined using the Evenson cut points for all children to ensure the dependent variable was comparable across all age groups. As many of the correlates of MVPA were skewed, and to compare the magnitude of the association across all variables, spearman rho correlations were computed. A Kruskal–Wallis test was used to test for differences across seasons. Analyses were stratified by MM and age (dichotomized into 2–9 years and 10–16 years of age). All statistical analyses were performed in IBM SPSS Version 25 (IBM SPSS Statistics, 2010) and significance levels were set at *p* < .05. Sample size calculations were completed for the planned longitudinal analyses of the MY LIFE study suggesting 80% power to detect non-linear trajectories of mental health over four waves of data collection.

## Results

### Descriptive Statistics

Children were on average 9.4 years old (*SD* = 4.2), and the majority were male (51.4%) and within the “normal” BMI range (66.4%). Most parents reported that both themselves (86.4%) and their child (95.0%) were born in Canada, they were White (87.1%), they were either married or in a common law relationship (86.4%), and their annual household income was below CAD$119,000 (52.1%) (Table 1).

Among children ages ≥ 5 years, 41.2% of the children with MM and 46.5% with PI met PA guidelines (see Table 2). Among children <5 years, no child with MM and 14.3% of children with PI met PA guidelines.

**Table 2.** Descriptive Statistics of PA Behaviors Stratified by Presence of Multimorbidity.

Variable	MM (*n* = 55)	PI (*n* = 85)
Meeting daily PA guidelines		
Ages 2 years (*n* = 5), *n* (%)	0 (0.0)	0 (0.0)
Ages 3–4 years (*n*=13), *n* (%)	0 (0.0)	2 (14.3)
Ages 5–16 years (*n* = 122), *n* (%)	21 (41.2)	33 (46.5)
Average daily min of MVPA		
<10 years of age (MM *n* = 29; PI *n* = 52), *M* (*SD*)	65.71 (20.13)	62.94 (17.31)
≥10 years of age (MM *n* = 26; PI *n* = 33), *M* (*SD*)	46.42 (21.05)	44.98 (19.19)
All ages (*n* = 140), *M* (*SD*)	56.59 (22.58)	55.97 (20.06)

*Note*. MM = multimorbidity; PI = physical illness only; PA = physical activity; MVPA= moderate-to-vigorous physical activity; *SD* = standard deviations; *n* = sample size.

### Correlational Analyses

Table 3 displays the correlations. Among children with MM, MVPA was significantly correlated with age, BMI percentile, child-rated self-perceived behavioral conduct, and parent-rated physical well-being and peer support health-related quality of life. The Kruskal–Wallis test showed that MVPA did not differ across seasons for children 2 to 16 years with MM (H = 3.52, *df* = 3, *p* = .318), or when stratified by age (<10 years: H = 2.03, *df* = 3, *p* = .566; and ≥10 years: H = 4.80, *df* = 3, *p* = .187). Among children with PI, age, sex, child-rated perceived social competence, child-rated perceived athletic competence, parent-rated physical health-related quality of life, frequency of participation in community sport, and family functioning were significantly correlated with MVPA. MVPA also differed by season for children with PI for all ages (H = 9.27, *df* = 3, *p* = .026), with the highest MVPA in the spring and lowest in the fall but not when stratified by age (<10 years: H = 4.72, *df* = 3, *p* = .193; and ≥10 years: H = 5.23, *df* = 3, *p* = .156).

**Table 3.** Bivariate Spearman Rho Correlations With Average Daily Minutes of MVPA.

Variable	MM			PI		
	All ages (*n* = 55)	<10 years (*n* = 29)	≥10 years (*n* = 26)	All ages (*n* = 85)	<10 years (*n* = 52)	≥10 years (*n* = 33)
Demographic and health						
Child age at baseline visit	−0.45**	−0.03	−0.28	−0.40**	−0.03	−0.21
Child sex (female)	−0.20	−0.05	−0.30	−0.26*	−0.22	−0.34
Parental relationship status	0.15	0.12	0.02	−0.06	−0.01	−0.17
Income greater than CAD$119,000	0.09	<0.01	0.11	0.01	−0.01	0.06
Child not born in Canada	−0.22	n/a	−0.14	−0.16	−0.21	−0.11
Body mass index percentile	−0.28*	−0.11	−0.35	−0.10	−0.01	−0.07
Parent total WHODAS score	−0.19	−0.21	−0.26	−0.12	−0.09	−0.21
Years since diagnosis of physical condition	−0.25	0.09	−0.23	−0.05	<.01	0.05
Psychosocial						
Parent SDQ Externalizing scale	0.25	0.04	0.28	0.08	−0.06	0.12
Parent SDQ Internalizing scale	−0.06	0.01	−0.09	−0.09	−0.08	−0.08
Child SPPC Scholastic Competence	0.04	n/a	0.04	0.01	n/a	0.01
Child SPPC Social Competence	0.11	n/a	0.11	0.42*	n/a	0.42*
Child SPPC Behavior Conduct	−0.45*	n/a	−0.45*	0.01	n/a	0.01
Child SPPC Athletic Competence	0.26	n/a	0.26	0.48**	n/a	0.48**
Child SPPC Physical Appearance	0.26	n/a	0.26	0.14	n/a	0.14
Child SPPC Global Self-Worth	−0.04	n/a	−0.04	−0.16	n/a	−0.16
Parent-Rated Physical HRQL	0.56**	0.44*	0.57**	0.34**	0.17	0.49**
Parent-Rated Psychological HRQL	0.22	0.26	0.01	0.10	−0.04	0.14
Autonomy & Parents HRQL	0.08	0.02	0.01	0.06	0.03	−0.08
Peers HRQL	0.27*	0.25	0.24	0.10	0.08	0.06
Parent-Rated School HRQL	−0.02	0.10	−0.28	−0.04	−0.08	−0.18
Family Assessment Device	0.18	0.11	0.16	0.25*	0.47**	0.10
Behavioral						
Frequency of Community Sport	0.21	n/a	0.21	0.41*	n/a	0.41*

*Notes*. MM = multimorbidity; PI = physical illness only; WHODAS = World Health Organization Disability Assessment Schedule; SDQ *=* Strengths and Difficulties Questionnaire; SPPC = Self-Perception Profile for Children; HRQL = health-related quality of life.

** *p* < .01. *p < .05.

## Discussion

This study aimed to quantify the average level of PA of both children with PI and MM and identify demographic, health, psychological/cognitive/emotional, behavioral, familial, and environmental variables that correlate with their PA. Approximately half of children in MY LIFE met the Canadian PA Guidelines. Compared with a nationally representative sample from the Canadian Health Measures Survey in 2012 and 2013, children in the MY LIFE study are engaging in approximately 5 to 13 fewer average minutes of daily MVPA across all age groups (Government of Canada, 2015). Similar findings from a recent systematic review of children with chronic physical diseases showed that while not markedly lower than children without illness, PA engagement was still insufficient (Elmesmari et al., 2017). Our study found that MVPA among children with and without multimorbidity was correlated with demographic, health, and psychosocial variables and a behavioural variable was also correlated to MVPA among children with a physical illness only.

Research among children with typical development consistently finds age, sex, and BMI correlated with MVPA (Government of Canada, 2015); however, only age was correlated to MVPA among both children with MM and PI. This is aligned with results of Mangerud and colleagues (2014) who found no effect of sex or absolute BMI among adolescents with mental illness. A 2016 systematic review found inconsistent effects of sex and no effect of BMI on PA of children with physical disabilities (Li et al., 2016). A possible explanation regarding the lack of covariation between MVPA and BMI may be that children with PI experience limitations at all body sizes (e.g., poor motor skills, fatigue, time). However, the sex imbalance among children with MM in this study (36.4% female) precludes ruling out an association with sex.

MVPA and parent-rated physical health-related quality of life were positively correlated among both groups, which is congruent with the research of Sawyer and colleagues (2004) that found adolescents with physical illness report that their health problems interfere with their participation in PA over time. Self-perceived behavioral conduct was correlated (negatively) with MVPA of children with MM. The SPPC behavioral conduct subscale asks children to reflect on how they perceive their ability to act in an appropriate manner, with lower scores indicating worse perceptions of behavioral conduct; children may be interpreting high-activity externalizing behaviors such as being restless, moving around the classroom, or being physically aggressive as inappropriate behavior. Therefore, lower perceptions of behavioral conduct may be reflecting higher levels of high-energy externalizing behaviors, which may explain the negative correlation with MVPA. Among children with PI, self-perceived social and athletic competence were positive correlates of MVPA, aligning with research observed in children with typical development (Poitras et al., 2016), which suggests that domains of self-concept that are related to sport or PA are often correlated with PA. Although likely bidirectional, children with higher perceptions of their athletic ability are often more motivated to continue pursuit of athletic activities and PA, and as PA is often a social activity among children, those with higher social perceptions of themselves are similarly likely to engage in PA (Bedard et al., 2020).

Similar to children with typical development, community sport, family functioning, and season were correlates among children with PI (Sallis et al., 2000; Schmutz et al., 2017). These results support the notion that community sport can provide opportunities for children to participate in organized PA and underscore the importance of positive family functioning. Parental support has consistently been associated with child PA (Beets et al., 2010), so it is possible that higher scores of family functioning may indicate increased parental support for PA, among other positive behaviors. Finally, seasonal differences in PA among children are often explained through time spent outdoors (Carson & Spence, 2010); therefore, the association between PA and season among children with PI may be due to limited time spent outdoor in the fall compared with the spring. These variables may not have been significant among children with MM because there are unknown barriers related to their MM that preclude their participation in leisure-time PA despite their engagement in community sport, positive family functioning, or season. Interestingly, peer support was positively correlated with MVPA only among children with MM suggesting that peer relationships and the feelings of support from friends may be an important factor for children with MM.

To our knowledge, this is the first study to objectively describe PA and its correlates among children with MM. The use of accelerometry to capture PA volume, the application of reliable and valid assessments of MM, and the generalizability of the sample across a range of physical illnesses provide considerable strength to our study. However, existing limitations must be acknowledged. First, just over half of the sample provided valid accelerometer data; therefore, analyses are likely underpowered and may be biased toward more physically active children. Second, the patterns of missing accelerometer data suggest that data may not be missing at random. Third, we are unable to qualify the type or context of PA in which children are engaging. Fourth, this study was designed primarily to answer questions relating to development of multimorbidity over time and did not include all potential correlates of PA nor a comparison sample of healthy children.

Overall, this study demonstrates that children with a physical illness, with or without multimorbidity, are insufficiently active and their PA engagement is correlated with variables at multiple levels of development. Future PA interventions for children with chronic illnesses may consider targeting older children through community sport/athletic programming to foster peer support and social competence. Programs should also consider constructing mastery motivational climates (Braithwaite et al., 2011; Robinson et al., 2009) to promote both the movement skills required for PA and perceptions of self given that various domains of self-perceptions were associated with PA among both children with PI and MM. Finally, as physical health-related quality of life was associated with PA for both children with PI and MM, family-centered care should continue to be prioritized (Moore et al., 2009). Prospective data are needed to explore the causal direction of these associations. Furthermore, it is essential to explore other unmeasured variables that may explain the variability in PA (e.g., motor skills and parental support for PA) and examine the patterns, different intensities, types, and context of PA as this will clarify the types of PA children feel able or unable to participate in.
